# The mediating role of life skills in the association of the coach–athlete relationship with youth athletes’ well-being

**DOI:** 10.1007/s44192-026-00468-7

**Published:** 2026-05-03

**Authors:** Willen Remon Tozetto, Claudio Olivio Vilela Lima, Fernando de Sousa Ferreira dos Santos, Natalie Durand-Bush, Michel Milistetd

**Affiliations:** 1 Federal University of Santa Catarina, Florianópolis, Brazil; 2 Instituto Politécnico do Porto, Porto, Portugal; 3 University of Ottawa, Ottawa, Canada; 4 Present Address: Florianópolis, Brazil

**Keywords:** Adolescence, Ddevelopment, Positive youth development, Mental health, Youth sport

## Abstract

The coach–athlete relationship is central for the development and well-being of adolescent athletes, but the mechanisms underlying this relation are underexplored. Learning life skills may explain these underlying mechanisms; therefore, understanding this line of inquiry can help optimize the coaching process and life skill development. Given this context, this study aims to analyze how the interactions between coaches and adolescent athletes are associated with well-being, as well as understand the statistical mediating role of life skills. A total of 312 Brazilian adolescent athletes participated, completing measures assessing the coach–athlete relationship, life skills and well-being. The paths (coach–athlete relationship → life skills → well-being) were tested using structural equation modeling. As a result, life skills showed a statistical indirect association accounting for 45.7% of the variance explained in the association between the coach–athlete relationship with well-being. Among the dimensions associated with the coach–athlete relationship, commitment emerged as a significant correlate of all life skills. Moreover, complementarity showed a direct association with all three well-being domains. Additionally, closeness showed a specific direct association only with psychological well-being. With regards to life skills, goal setting was positively associated with all three well-being domains, whereas emotional skills showed a negative association, suggesting that they may function as a marker of prior adversity. Taken together, the coach–athlete relationship is not a monolithic construct; its dimensions show functional specificity in their associations. From an applied standpoint, sport-based programs should prioritize building a relationship of commitment as a foundational element to support the intentional teaching of skills.

**Supplementary Information:**

The online version contains supplementary material available at 10.1007/s44192-026-00468-7.

## Introduction

Sport participation is a global phenomenon and one of the most popular organized activities among adolescents in Brazil [1], being recognized for its potential to promote positive youth development (PYD) [2, 3]. However, there are emerging challenges that can negatively impact PYD such as significant dropout rates among adolescents (20 to 30% per year), which are frequently associated with a lack of satisfaction of basic psychological needs and self-determined motivation [4]. To overcome such challenges, coaches are among the main agents responsible for structuring practice environments and their actions directly influence youths’ long-term engagement in sports [4].

Previous studies indicate that coach effectiveness lies less in isolated procedural knowledge and more in the quality of the dyadic relationships they establish with the athlete [4, 5]. This emphasis on coach–athlete relationships reflects a relational and interdependent approach toward coach effectiveness [6]. To analyze the structural dimensions of the coach–athlete relationship, the 3 C model proposed by Jowett [5] offers a theoretical framework focused on the interdependence of coaches’ and athletes’ feelings, thoughts and behaviors. This model is operationalized through three domains: closeness (affective), which reflects the bond of trust and respect between coaches and athletes; commitment (cognitive), the intention to maintain a long-term partnership; and complementarity (behavioral), which refers to the effectiveness of coach–athlete interactions during sport practice [5]. Thus, the quality of coach–athlete relationships is defined by these components, which can induce meaningful psychosocial outcomes such as PYD and life skills.

Life skills can be positioned as functional and transferable competencies that individuals use to manage the demands of life [3]. For example, competencies such as leadership and communication, which include active listening and clear expression, can be developed in sport and effectively transferred to academic and/or other life domains. Sport programs are conducive environments for life skill development as coaches can facilitate learning implicitly as an automatic byproduct of practice, as well as in an explicit and intentional manner [3, 7]. Typically, most research has focused on youth’s developmental processes, however recent discussions and frameworks suggest that life skill development and transfer is contingent on culture, social norms, politics, and many other environmental factors [8, 9]. Such perspective towards life skill development highlights the need to situate life skills in a broader system of relations and help youth contribute to positive social change [8, 9]. For any approach to be effective, the existence of a quality relational environment is a fundamental prerequisite [10]. However, the coach’s influence appears to be more prominent for certain life skills (e.g., teamwork, goal setting) than for others, which may be facilitated by parents or teammates [11]. Thus, life skills function as psychological tools that allow athletes to interact more adaptively in sport and across life domains, thereby impacting their well-being [2, 12, 13].

Well-being is therefore a fundamental indicator of PYD and can contribute to continued participation in sport [13, 14]. Well-being is understood through the multifaceted model advanced by Keyes and adapted for sport in Portuguese by Tozetto et al. [14], which considers dimensions such as emotional well-being (satisfaction and positive affect); social well-being (integration and a sense of belonging to the team); and psychological well-being (personal growth, autonomy, and purpose in life)—all of which are essential components of flourishing. The literature supports that the quality of the coach–athlete relationship is directly associated with well-being. Specifically, a high-quality relationship is associated with positive well-being. Conversely, a low-quality relationship is associated with negative outcomes such as burnout, anxiety and depression [15]. Moreover life skills can also contribute to well-being, with a focus on the development of youth assets rather than on deficits [2].

## The present paper

The present study is grounded in the PYD framework [2, 3], which positions sport as a context for strengthening internal assets, with well-being as its fundamental indicator of success [13]. For PYD to occur, the quality of the coach–athlete relationship operationalized in the present study by the 3Cs Model (closeness, commitment, and complementarity) [5] serves as the primary quality indicator [6]. According to the Self-Determination Theory (SDT), a high-quality relational environment satisfies athletes’ basic psychological needs for autonomy, competence, and relatedness [10, 16], creating the conditions for them to internalize functional life skills [17]. Thus, life skills act as a mediating mechanism [2, 12, 13] that translates relational support into human flourishing [6] manifested in the emotional, social, and psychological domains of well-being [14, 15].

The importance of investigating how sports can foster PYD, life skills, positive coach–athlete relationships and well-being becomes critical given the challenging landscape of youths’ mental health. In Brazil, for example, mental health problems affect 19.2% of young people up to 19 years of age [18]. Hence, the need for environments that promote well-being is critical as athletes often feel seen only for their performance and not as a ‘complete and unique person’ [6]. Additionally, the literature suggests that well-being may be a prerequisite for success in sports, which also makes this line of inquiry relevant [6]. Although the literature recognizes the importance of the coach–athlete relationship and well-being, studies that empirically test a model that positions life skill development as the mediating mechanism through which the coach–athlete relationship influences the different domains of well-being are lacking. Understanding these processes could help optimize youth sport programming, particularly coaching practices and coach education.

Therefore, the present study aims to analyze how the interactions between coaches and adolescent athletes are associated with well-being and to understand the statistical mediating role of life skills. These findings may provide an empirical basis for the delivery of youth sport programming. The theoretical rationale for the directional hypotheses formulated in the present study is predicated on the specialized functions of the 3Cs dimensions within a multivariate framework [5, 19]. Although closeness represents the affective bond and emotional warmth of the coach–athlete relationship, which are dimensions traditionally linked to general well-being, this study hypothesizes that closeness might primarily serve as a relational secure base in the context of skill acquisition [20]. Rather than acting as a direct enabler of life skill acquisition, closeness is posited as a potential prerequisite for the developmental process [20, 21]. Specifically, within adolescent sport, the coach’s commitment, defined as the cognitive intention to maintain the partnership and invest in the athlete’s future is hypothesized to be more salient for the internalization of goal-oriented competencies [19]. Moreover, complementarity is posited to primarily predict skills requiring behavioral coordination and social interaction such as teamwork and social skills reflecting the quality of interpersonal coordination during practice [5, 11]. Thus, by testing these dimensions simultaneously, the present study seeks to identify the unique relative contribution of each component, acknowledging that a robust affective bond allows active relational forces to potentially exert a more direct influence on the athlete’s developmental trajectory [19, 20].

The specific hypotheses of the study are as follows: (1) closeness is hypothesized to be directly associated with emotional and social well-being, while potentially showing a non-significant association with psychological well-being or life skill acquisition when other relational dimensions are present; (2) commitment is predicted to be associated with interpersonal communication, teamwork, social skills, and leadership; (3) complementarity is expected to be associated with goal setting, problem solving and decision making, emotional skills, and time management; and (4) consistent with previous evidence [11], it is hypothesized that only a specific subset of life skills, such as teamwork, goal setting, social skills, and problem solving and decision making, will be significantly associated with well-being outcomes.

## Method

### Study design

This study followed a cross-sectional design with a sample of athletes from different sports across two competitive youth sport programs. The methodology was quantitative with variables collected through validated questionnaires that assessed the coach–athlete relationship, life skills and well-being.

Data collection took place between July 2023 and December 2023. Researchers sent emails to the tutors or parents of potential participants with project information alongside the questionnaires administered via an online form (i.e., google forms). The eligibility criteria were as follows: (a) participants aged between 12 and 17 years; and (b) participants that had regular involvement in organized youth sport (i.e., minimum of two practices per week). This age range reflects the standard age group for secondary school students in Brazil.

### Context

The Brazilian sport landscape is characterized by a contrast between professionalized elite clubs in metropolitan hubs and underfunded municipal programs in smaller cities, depicting distinct contexts for athletic participation [22, 23]. Accordingly, the study sample was conveniently selected from two distinct sports contexts to reflect aspects of the diversity of the Brazilian sports ecosystem, where social and cultural structures are linked to different opportunities for young athletes’ participation and inherently development [24]. This sampling strategy aimed to illustrate the contrast between high-performance hubs and resource-limited programs, which are associated with different developmental experiences and outcomes [25]. The first is a high-performance sports club located in a large metropolitan area with an estimated population of 2.32 million people, recognized for developing Olympic athletes. This environment is characterized by a fully professional structure, comprising specialized facilities and multidisciplinary teams (including sports psychologists, physiotherapists and nutritionists). In contrast, the second context consists of three municipal youth sport programs across three small cities (with a combined population of 103,000 people). These programs operate with limited resources, shared multi-sport facilities, and a single coach responsible for delivering the program across different age groups.

### Sample

This study is part of a larger study, and the sample size (*N* = 312) substantially exceeds that recommended for the mediation model tested. Adopting a conservative assumption of medium magnitude effects (β ≈ 0.39) and using the Sobel test as a reference, the sample required to achieve 80% statistical power would be 90 participants [26].

Data from 312 athletes were analyzed, 248 athletes from a club located in a large city and 64 athletes from small cities in the same microregion. Regarding the large-city sample, 144 (58.1%) youth athletes had competed at least once in national or international competitions. In contrast, only nine (14.1%) athletes from the small-city sample had competed at the same level with the vast majority participating exclusively in municipal and/or regional events. The sports practiced by the adolescents were as follows: 74 (23.7%) futsal, 112 (35.9%) volleyball, 36 (11.5%) swimming, 27 (8.7%) tennis, 20 (6.4%) judo, 27 (8.7%) basketball, 14 (4.5%) artistic gymnastics, and two (0.6%) trampoline gymnastics. Table 1 presents descriptive information for the athletes.

### Data collection instruments

In the present study, three instruments were used. The coach–athlete relationship questionnaire (CART-Q), particularly the version validated by Vieira et al. [27] was used. The instrument consists of 11 items distributed across three domains: closeness (4 items), commitment (3 items), and complementarity (4 items). Each item is rated on a seven-point Likert scale ranging from 0 (strongly disagree) to 6 (strongly agree).

Additionally, life skill development was measured via the life skills scale for sport (LSSS) in its version validated for the Brazilian context by Nascimento-Junior et al. [28]. The instrument is composed of 43 items distributed across eight distinct domains: teamwork, goal setting, social skills, problem solving and decision making, emotional skills, leadership, time management, and interpersonal communication. Each item is answered on a five-point Likert scale ranging from 0 (not at all) to 4 (extremely).

Finally, the mental health continuum in sport—short version (S-MHC) in its version validated for Portuguese by Tozetto et al. [14] was also used. This instrument is composed of 14 items that assess three domains of well-being: emotional, social, and psychological. The items are distributed as follows: the emotional domain is measured by items 1–3; the social domain is measured by items 4–8; and the psychological domain is measured by items 9–14. Each item is answered on a six-point frequency scale ranging from 0 (never) to 5 (every day). The total score is calculated as the mean of the responses.

In all questionnaires, higher values indicate positive outcomes for the domains. Internal consistency for the instruments was assessed using McDonald’s omega (ω). The CART-Q showed good reliability (ω = 0.857; 95% CI 0.833–0.881]), as did the S-MHC (ω = 0.866; 95% CI 0.844–0.888]). The LSSS demonstrated excellent internal consistency (ω = 0.970; 95% CI 0.965–0.975).

The variables of sex (female = 0; male = 1); age (in complete years); region (large city = 0; small cities = 1), type of sport (team = 0; individual = 1) and time practicing the sport (in complete years) were used as covariates to control for potential confounding effects.

### Procedures and ethical considerations

The research received a favorable opinion (n. 5.955.858) from the Ethics Committee for Research with Human Beings at the Federal University of Santa Catarina (CEPSH-UFSC). The study was conducted in accordance with Resolution 466/12 of June 12, 2012, from the National Health Council/Brazil and the Helsinki Declaration. All participants were properly informed about the procedures. Informed consent was obtained from all individual participants included in the study, as well as from their legal guardians.

### Data analysis

The data were analyzed via RStudio data analysis software, version 2025.05.0. The data was imputed using the MICE package and the Predictive Mean Matching (PMM) method. Structural Equation Modeling (SEM) from the *lavaan* package was employed to understand the paths between variables. Three models were tested that followed the same logical model: coach–athlete relationship → life skills → well-being. The variables were constructed as second-order latent variables. The first level consisted of the questionnaire items loading onto the latent domains, and the second level consisted of the latent domains loading onto a general factor for each questionnaire. For Model 1, the path was tested among the second-order general factors. For Model 2—Initial, the logical path was tested among the first-order latent domains, without specifying the second-order level. In this model, the three latent domains of the Coach–Athlete Relationship, the eight life skills domains, and the three well-being domains were used to test the proposed logical sequence. A backward elimination method was subsequently used to remove nonsignificant paths and to achieve a more parsimonious model, which was designated Model 3—Final. The control variables were included in all the models. The R script for the models is presented in Supplementary Material 1.

The fit indices included the following criteria and cutoff points: degrees of freedom (df) equal to or greater than 1 and a χ²/df ratio less than 3.00. A Comparative Fit Index (CFI) and Tucker–Lewis Index (TLI) above 0.9 indicate acceptable fit. The Root Mean Square Error of Approximation (RMSEA) and Standardized Root Mean Square Residual (SRMR) were used, with values below 0.08 indicating an acceptable fit and values between 0.08 and 0.10 indicating a reasonable fit [29]. The significance level adopted was *p* < 0.05.

## Results

Table 1 summarizes the sample characteristics and comparisons between the collective (*n* = 213) and individual (*n* = 99) sport athlete groups. No statistically significant differences were observed between the groups regarding sex distribution (*p* = 0.127) or age (*p* = 0.783). A comparison of the scores for the general domain did not reveal significant differences between the groups for well-being (*p* = 0.726), life skills (*p* = 0.540), or coach–athlete relationship (*p* = 0.070).

**Table 1:** General information about athletes across types of sport

Categorical variables	Team (*n* = 213)	Individual (*n* = 99)
	*n* (%)	*n* (%)
Sex		
Male	110 (51.6%)	61 (61.6%)
Female	103 (48.4%)	38 (38.4%)

Continuous variables	$\:\stackrel{-}{\mathrm{x}}$ ± SD	$\:\stackrel{-}{\mathrm{x}}$ ± SD
Age (in full years)	14.2 ± 1.74	14.2 ± 1.90
Well-being	4.27 ± 0.53	4.25 ± 0.55
Emotional well-being	4.26 ± 0.61	4.18 ± 0.59
Social well-being	4.25 ± 0.68	4.27 ± 0.69
Psychological Well-being	4.30 ± 0.49	4.28 ± 0.64
Life skills	3.09 ± 0.61	3.04 ± 0.69
Teamwork	3.31 ± 0.60	3.04 ± 0.73*
Goal setting	3.25 ± 0.71	3.31 ± 0.76
Social skills	3.03 ± 0.74	2.80 ± 0.88*
Problem solv. and decision making	2.97 ± 0.86	2.97 ± 0.84
Emotional skills	3.05 ± 0.81	3.14 ± 0.77
Leadership	3.10 ± 0.69	2.98 ± 0.80
Time management	2.95 ± 0.85	3.20 ± 0.80*
Interpersonal communication	3.07 ± 0.74	2.90 ± 0.92
Coach–athlete relationship	6.40 ± 0.61	6.52 ± 0.51
Closeness	6.66 ± 0.59	6.71 ± 0.49
Commitment	6.03 ± 0.96	6.30 ± 0.71
Complementarity	6.52 ± 0.59	6.56 ± 0.57

$\:\stackrel{-}{\mathrm{x}}$ = Mean; N = Absolute frequency; % = Relative frequency; SD = Standard deviation; Solv. = Solving; * = Significant difference at the *p* < 0.05 level

Table 2 presents the fit indices for the three structural models tested. Model 1-Basic showed a good overall fit. Model 2-Initial showed some good fit indices (χ²/df = 1.91; RMSEA = 0.059), but the standardized root mean square residual was slightly above the cutoff point (SRMR = 0.099), and notably, the comparative fit indices (CFI = 0.809; TLI = 0.796) were below the reference values. Model 3-Final, resulting from the backward elimination procedure (*p* < 0.05), demonstrated a reasonable fit to the data. While the absolute fit indices (χ²/df = 2.41; RMSEA = 0.067) were adequate, the incremental fit indices (CFI = 0.881; TLI = 0.874) and the residual indices (SRMR = 0.121) remained borderline, falling slightly below conventional thresholds for a good fit. These values indicate that the final model is a parsimonious approximation of the theoretical structure. Therefore, the coefficients of Model 2 are not presented, and the results focus on the Model 3 acknowledging the limitations in its global fit indices.

**Table 2:** Fit indices for the models

	df	χ²	χ²/df	CFI	TLI	RMSEA (95% IC)	SRMR
Model 1—Basic	2326	3256	1.40	0.958	0.959	0.036 (0.033/0.039)	0.064
Model 2—Initial	2258	4311	1.91	0.809	0.796	0.059 (0.056/0.061)	0.099
Model 3—Final	1305	3146	2.41	0.881	0.874	0.067 (0.064/0.070)	0.121

df = degrees of freedom. χ² = chi-square test. TLI = Tucker – Lewis index. CFI = comparative index. RMSEA = root mean square error of approximation. SRMR = standardized root mean square residual

**Fig. 1:**
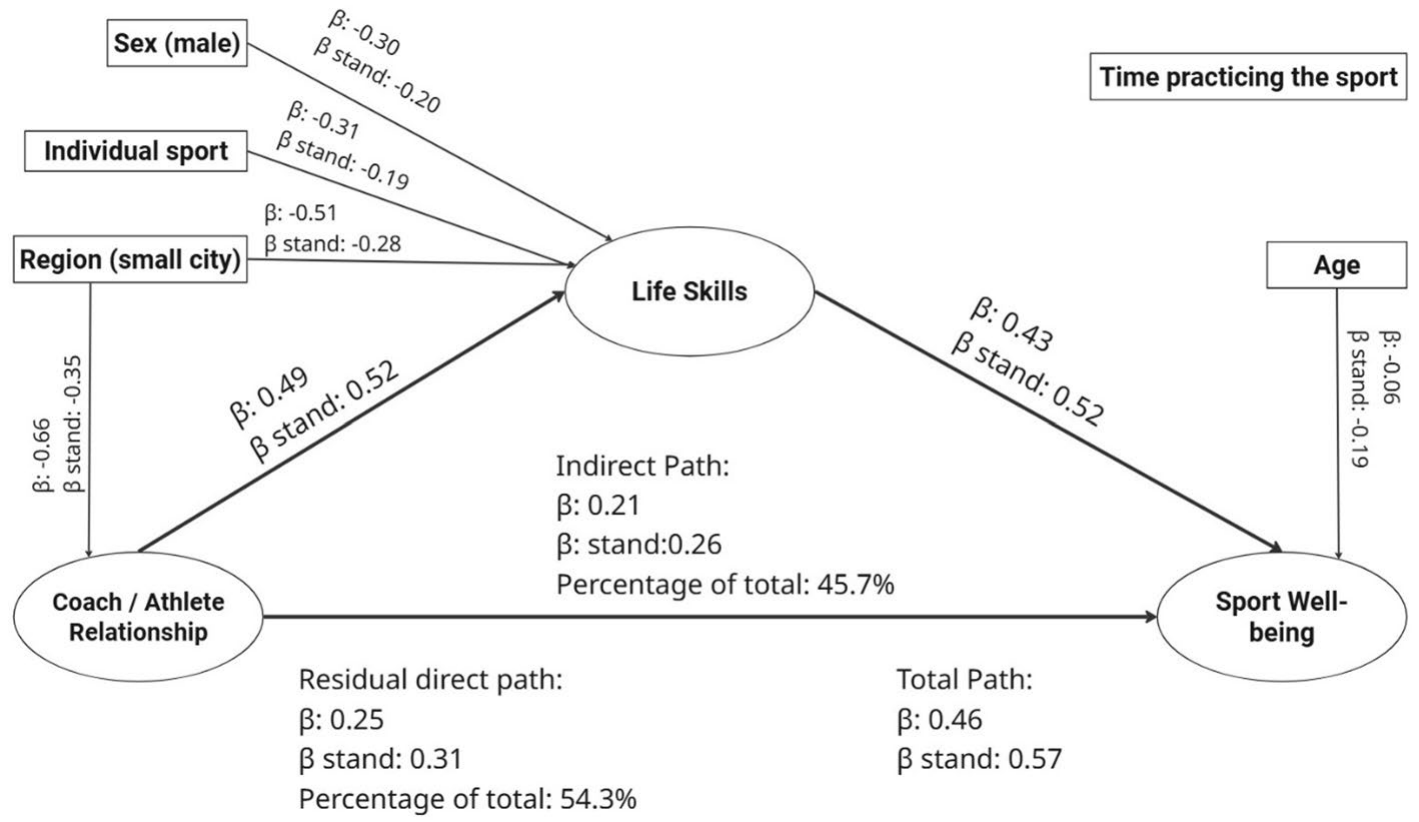
Graphical representation of the significant paths for the Model 1—Basic. β = non standardized coefficient. β Stand = Standardized coefficient. All paths were significant (*p* < 0.05). The analysis was adjusted for sex, age, region, type of sport and time spent practicing the sport

Table 3 presents the direct and indirect associations for Model 1. The coach–athlete relationship was positively and significantly associated withlife skills, indicating that a higher quality relationship is linked togreater life skills (β = 0.49; stan. β = 0.52). In turn, life skills showed a direct and positive association with well-being (β = 0.43; stan. β = 0.52). Even after accounting for the indirect pathway through life skills, the direct path from the coach–athlete relationship to well-being (path c’) remained positive and statistically significant (β = 0.25; stan. β = 0.31). The indirect path mediated by life skills (β = 0.21; stan. β = 0.26) accounted for 45.7% of the total variance explained. Figure 1 graphically represents the presented model.

**Table 3:** Direct and indirect paths through the structural equation model between the analyzed variables at model 1

Direct paths	β	95% CI	SE	β stan.	*P* value
Well-being					
Life Skills (b)	0.43	0.31/0.54	0.06	0.52	< 0.001
Coach–athlete relationship (c’)	0.25	0.16/0.34	0.05	0.31	< 0.001
Life skills					
Coach–athlete relationship (a)	0.49	0.38/0.60	0.06	0.52	< 0.001

Indirect paths	β	95% CI	SE	β stan.	% of β	% of β stand.
CART → Life Skills → Well-Being (a*b)	0.21	0.13/0.29	0.04	0.26	45.7%	45.7%
Total Path ((a*b) + c’)	0.46	0.34/0.58	0.06	0.57	–	

Percentage for indirect path represents the proportion of the total effect on well-being outcome explained by the mediator. β = Non standardized coefficient. β Stand = Standardized coefficient. a*b = Indirect path effect. c’ = Residual direct path effect. c’ + (a*b) = Total path effect. Analysis adjusted for sex, age, region, type of sport and time practicing the sport

**Fig. 2:**
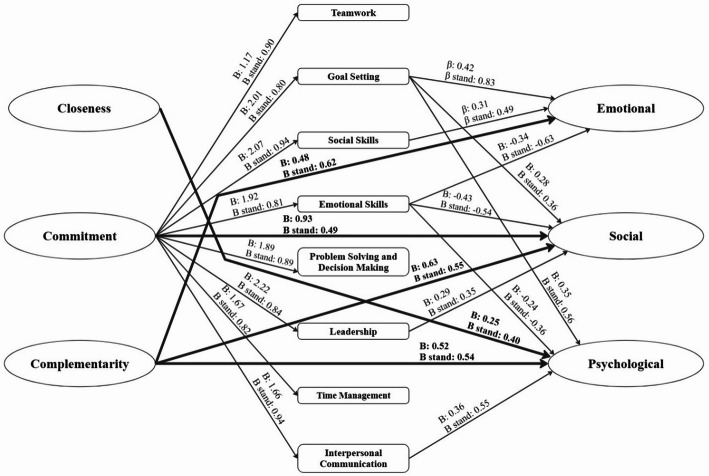
Graphical representation of the significant paths for the Model 3—Final. β = Non standardized coefficient. β Stand= Standardized coefficient. Only significant paths were plotted (*p* < 0.05). Dark paths are direct paths from coach–athlete relationship to well-being. Light paths are paths that pass-through life skills. The analysis was adjusted for sex, age, region, type of sport and time spent practicing the sport

Figure 2 presents Model 3—Final. It reports the associations among the dimensions of the coach–athlete relationship (closeness, complementarity, and commitment), the eight life skills, and the well-being dimensions (emotional, social, and psychological). All paths presented in the model are statistically significant (*p* < 0.05). The detailed values are presented in the table in Supplementary Material 2.

When the closeness dimension was analyzed, only one direct, positive, and significant association with psychological well-being (β = 0.25; stan. β = 0.40) was observed. Closeness did not show other direct associations on well-being or on life skills. In terms of complementarity, positive and significant associations with emotional well-being (β = 0.47; stan. β = 0.62), social well-being (β = 0.62; stan. β = 0.55), and psychological well-being (β = 0.51; stan. β = 0.54) were identified. The commitment dimension showed a direct and positive association with social well-being (B = 0.93; β = 0.49) and on the other eight life skills (ranging from β = 1.17; stan. β = 0.90 to β = 2.22; stan. β = 0.84). Importantly, the positive and significant paths between commitment and all eight life skills were consistent across both the Model 2 and Model 3 models, confirming the robustness of this dimension as a primary predictor.

Among the paths linking the eight life skills to well-being, five were statistically significant. The skills of teamwork, problem solving and decision making, and time management did not show significant associations (*p* > 0.05). Among the statistically significant life skills, goal setting was positively associated with all three well-being dimensions: emotional (B = 0.42; β = 0.83), social (B = 0.28; β = 0.36), and psychological (B = 0.35; β = 0.56). In contrast, emotional skills were also associated with all three dimensions but showed a negative correlation with lower levels of emotional (B = − 0.34; β = − 0.63), social (B = − 0.43; β = − 0.54), and psychological (B = − 0.24; β = − 0.36) well-being. Notably, these negative associations remained stable and consistent in sign and magnitude from the saturated model (Model 2) to the final model (Model 3).

The remaining life skills showed specific associative paths to the well-being domains. Social skills were positively associated with only emotional well-being (B = 0.31; β = 0.49). Similarly, leadership was linked to social well-being (B = 0.29; β = 0.35), whereas interpersonal communication significantly correlated with only psychological well-being (B = 0.36; β = 0.55).

Table 4 details the indirect paths from the commitment dimension to emotional well-being. The association between commitment and emotional well-being showed positive indirect associations through goal setting (β = 0.85; stan. β = 0.67) and social skills (β = 0.65; stan. β = 0.47). In contrast, emotional skills showed a negative indirect association (β = -0.65; stan. β = -0.51). The contribution of each path to the total association, in order of magnitude, was identified for goal setting (β = 87%; stan. β = 82%), followed by emotional skills (β = − 67%; stan. β = − 63%) and social skills (β = 66%; stan. β = 57%). The direct path (c’) between commitment and emotional well-being was not statistically significant (β = 0.14; stan. β = 0.19), representing a small portion of the total association (β = 14%; stan. β = 23%).

For social well-being, the association with commitment showed positive indirect paths through goal setting (β = 0.56; stan. β = 0.30) and leadership (β = 0.64; stan. β = 0.30). On the other hand, emotional skills showed a negative indirect association (β = − 0.83; stan. β = − 0.44). The relative contribution of each path to the total association, in order of magnitude, was identified for emotional skills (β = − 65%; stan. β = − 69%), followed by leadership (β = 50%; stan. β = 47%) and goal setting (β = 43%; stan. β = 46%). The direct path (c’) between Commitment and Social well-being was statistically significant (β = 0.93; stan. β = 0.49), representing the largest portion of the total association (β = 72%; stan. β = 76%).

The association between commitment and psychological well-being was identified through three indirect paths. The goal setting (β = 0.71; stan. β = 0.45) and interpersonal communication (β = 0.93; stan. β = 0.52) showed positive indirect associations, whereas emotional skills showed a negative indirect association β = − 0.46; stan. β = − 0.30). The relative contribution of each path to the total association, in order of magnitude, was identified forinterpersonal communication (β = 80%; stan. β = 78%), followed by goal setting (β = 61%; stan. β = 68%) and emotional skills (β = − 40%; stan. β = -45%). The direct path (c’) between commitment and psychological well-being was not statistically significant (β = − 0.02; stan. β = − 0.01) and showed a minimal contribution to the total association (β = − 2%; stan. β = − 2%).

The direct associations show the specific contributions of the closeness and complementarity dimensions to the overall model associations (total path for all). In terms of magnitude, the largest direct contribution came from complementarity with respect to emotional well-being (β = 9%; stan. β = 13%). Next, complementarity showed similar associations with social well-being (β = 12%; stan. β = 11%) and psychological (β = 10%; stan. β = 11%) well-being. The final direct association came from the link between closeness and psychological well-being (β = 5%; stan. β = 8%). In an aggregated analysis, the total indirect association of Model 3 (β = 45%; stan. β = 43%) to overall well-being was comparable in magnitude to that of Model 1 (β = 42.6%; stan. β = 42.7%).

**Table 4:** Direct and indirect effects of the coach–athlete relationship on sports well-being via life skills for model 3

Paths	β	95% CI	SE	β stan.	% of β	% of β stand.
Commitment → goal setting→ emotional WB (a*b)	0.85	0.39/1.32	0.24	0.67	87%	82%
Commitment → emotional skills → emotional WB (a*b)	− 0.65	− 1.10/− 0.21	0.23	− 0.51	− 67%	− 63%
Commitment → social skills → emotional WB (a*b)	0.65	0.21/1.08	0.23	0.47	66%	57%
Commitment → emotional WB (c’)	0.14	− 0.05/0.33	0.10	0.19	14%	23%
Total path to emotional WB (a*b + c’)	0.98	0.47/1.25	0.20	0.82	41%	38%
Commitment → goal setting→ social WB (a*b)	0.56	0.19/0.93	0.19	0.30	43%	46%
Commitment → emotional skills → social WB (a*b)	− 0.83	− 1.25/− 0.41	0.21	− 0.44	− 65%	− 69%
Commitment → leadership → social WB (a*b)	0.64	1.43/2.68	0.32	0.30	50%	47%
Commitment → social WB (c’)	0.93	0.25/1.60	0.35	0.49	72%	76%
Total path to social WB (a*b + c’)	1.29	0.85/1.82	0.25	0.65	54%	30%
Commitment → goal setting → psychological WB (a*b)	0.71	0.37/1.05	0.17	0.45	61%	68%
Commitment → emotional skills → psychological WB (a*b)	− 0.46	− 0.76/− 0.17	0.15	− 0.30	− 40%	− 45%
Commitment → inter. commu. → psychological WB (a*b)	0.93	0.49/1.37	0.22	0.52	80%	78%
Commitment → psychological WB (c’)	− 0.02	− 0.69/0.66	0.35	− 0.01	− 2%	− 2%
Total path to psychological WB ((a*b) + c’)	1.16	0.71/1.65	0.24	0.67	49%	31%
Total indirect path for commitment to WB (a*b)	2.39	2.15/4.59	0.62	2.13	45%	43%
Closeness → psychological WB (c’)	0.25	0.17/0.34	0.04	0.40	5%	8%
Complementarity→ emotional WB (c’)	0.48	0.29/0.66	0.10	0.62	9%	13%
Complementarity → social WB (c’)	0.63	0.42/0.83	0.10	0.55	12%	11%
Complementarity → psychological WB (c’)	0.52	0.34/0.69	0.09	0.55	10%	11%
Total Path for all (a*b + c’)	5.31	3.84/6.65	0.72	4.93	–	–

Percentages for indirect paths represent the proportion of the total effect on each well-being outcome explained by the mediator. Percentages for the total path of each specific well-being dimension indicate their respective contribution to the total effect on general well-being. Inter. Comm. = Interpersonal Communication. β = Nonstandardized coefficient. β Stand= Standardized coefficient. Only significant paths were calculated (*p* < 0.05). a*b: Indirect path effect. c’: Residual direct path effect. c’ + (a*b): Total path effect. The analysis was adjusted for sex, age, region, type of sport and time practicing the sport

## Discussion

This study aimed to analyze how the interactions between coaches and adolescent athletes are associated with well-being and to understand the statistical mediating role of life skills. The main findings reveal that life skills explain 45.7% of the variance in the association between the coach–athlete relationship and well-being. Closeness with the coach showed a positive association with only psychological well-being, and complementarity was positively associated with all three well-being domains. Furthermore, the commitment domain, in addition to having a direct association with social well-being, showed a positive link to the perceived learning of all eight life skills. However, only goal setting and emotional skills were associated with all three domains of well-being, with the former showing a positive correlation and the latter a negative one. Additionally, social skills showed a significant associative path to emotional well-being, leadership to social well-being, and interpersonal communication to psychological well-being.

### Life skills as mediators

The results of the present study indicate that the coach–athlete relationship is an important construct linked to well-being, with indirect associations through life skills. This structure is consistent with the literature, which indicates that a climate of support, trust, and cooperation helps to satisfy athletes’ basic psychological needs, supporting their self-determined motivation and the development of life skills [10, 16]. In turn, the development of life skills can foster moral values and a sense of competence [17], potentially allowing the athlete’s ability to flourish in sports [6], which may be linked to higher levels of well-being.

Additionally, a residual direct association between the coach–athlete relationship and well-being was observed. Other studies have also identified this link, albeit through different mediating mechanisms [5]. Despite the different pathways (life skills vs. motivational processes), the convergence of multiple studies on a partial mediation model reinforces the robustness of the direct link of the coach–athlete relationship on athlete well-being. One explanation for this direct associative path is that a high-quality relationship, in itself, is associated with well-being, potentially through its link to the satisfaction of athletes’ basic psychological needs [5].

However, the premise that a high-quality coach–athlete relationship is a universally achievable starting point must be problematized, as intrinsic factors of the athlete may represent barriers. Athletes with avoidant attachment often resist affiliations [20], which may be associated with difficulties in the development of an effective coach–athlete relationship and potentially linked to fewer possibilities for the development of life skills. Overcoming this barrier may involve deliberate effort from the coach to create opportunities for connection, a challenge that transcends conventional coaching practices (e.g., focusing primarily on technical instruction, tactical strategy, and performance outcomes over relationship building) [20].

Analysis of the confounding variables showed that while men have lower overall life skills than women do, the literature suggests that these differences may be domain specific. According to the findings of Ilham et al. [30], the differences by gender vary by specific competence: women score higher in time management and emotional and social skills, whereas men show higher scores in communication and leadership [30]. This lower overall baseline in men may explain why the review by Bruner et al. [2] found that interventions are associated with greater changes in men, as they may present more room for improvement.

Our finding that older athletes report greater well-being contradicts some previous literature, suggesting that well-being is not necessarily lower as competitive pressures rise with age [31]. This discrepancy is potentially linked to our study’s unique focus on well-being as an outcome associated with the coach–athlete relationship and life skills. From this perspective, the positive association between age and well-being may be related to the cumulative time athletes experience to acquire skills and cultivate stronger relationships. This interpretation aligns with the bioecological model in sport [24], which suggests that prolonged engagement fosters adaptation and positive social connections, ultimately enhancing well-being.

Individual sports are associated with lower life skills than are team sports, a finding that may be attributed to the distinct social interaction and teamwork inherent in team environments [32, 33]. The team environment is associated to the development of interpersonal skills such as leadership and communication, offering coaches a more favorable context for teaching these competencies [32–34]. In contrast, individual athletes tend to focus more on self-management and goal achievement than on enjoyment [32, 34].

Our results indicated that athletes from small cities reported lower scores for both the coach–athlete relationship and life skills when compared to athletes from the large city club. This difference can be understood through the contextual disparities between the investigated sport environments. In the Brazilian context, large urban centers have a greater concentration of financial resources, which is associated with the existence of clubs with multiprofessional structures [22], a reality reflected in one of the settings analyzed in this study. In contrast, smaller municipalities and rural areas offer a more restricted range of modalities, are financed by city halls with scarce resources, and are influenced by a limited number of coaches [22]. Therefore, the disparity in resources and professional support structures is potentially linked to the observed differences in relational quality and life skills development.

### Direct association of closeness with psychological well-being

Our results indicate a positive association between closeness in the coach–athlete relationship and psychological well-being. This finding corroborates the perspective that the coach can act as a trustful role model, where the quality of the relationship itself represents a protective factor for the individual [20]. A relationship of closeness and trust can provide the necessary secure base for the athlete to feel supported and valued, which are elements that are central to psychological well-being.

Notably, the absence of associations from closeness to life skills, as well as towards emotional and social well-being constitutes a striking feature of our final model. Rather than a mere descriptive absence, this lack of association suggests that in our sample the affective bond (closeness) functions as a specialized foundational pillar for individual psychological health rather than a broad catalyst for other domains such as commitment. This finding challenges the traditional prominence of closeness within the 3Cs framework, where this is often expected to be the strongest predictor of social and affective outcomes [5]. In our sample, however, closeness appears to have a “domain-specific” limitation. While closeness provides the affective security necessary for personal growth and purpose (psychological well-being), immediate emotional satisfaction and affect (emotional well-being) in a competitive context may be more closely tied to the fluid and goal-oriented cooperation of complementarity. Similarly, the lack of association with social well-being suggests that the bond is primarily dyadic; while it strengthens the athlete’s individual sphere, it may not necessarily extend to broader perceptions of social integration or team belonging, which appear to rely more on the long-term stability offered by commitment.

Our results reinforce this distinction, suggesting that without this relational base, the formal efforts towards teaching life skills may not lead to the internalization of life skills, consistent with the absence of mediation observed. This aligns with PYD frameworks that distinguish the PYD climate from an explicit life skill focus [35]. As illustrated by the “pyramid of teaching success” based on the coach John Wooden [21], relationship qualities such as closeness, including friendship form the foundation upon which more instrumental competencies are subsequently built. Furthermore, the lack of association with emotional well-being merits consideration of the complexity of the coach–athlete relationship. As pointed out by McGee and DeFreese [36], closeness can be simultaneously associated with positive (engagement) and negative (burnout) outcomes, potentially nullifying a linear association. This suggests a potential nonlinear or U-inverted relationship where moderation is essential, a concept reinforced by the “Balance” pillar in the Pyramid of Success [21]. Within this framework, Balance is defined as the maintenance of mental and emotional equilibrium staying grounded and avoiding the instability of emotional extremes. In the coaching dyad, this implies that the benefits of closeness are contingent upon a balanced state; an excessive or unbalanced bond may lead to emotional exhaustion or loss of objectivity, whereas a balanced closeness provides the stability necessary for psychological growth [21]. Therefore, closeness seems to function more as a foundational pillar for psychological health than as a direct catalyst for other skills or well-being domains in the context of our sample.

### Direct association of complementarity with well-being

The findings demonstrated that complementarity showed a positive and consistent association with all three well-being domains (psychological, social, and emotional). The consistency of this finding suggests that complementarity, which reflects the cooperation and effectiveness of the dyad, is characterized by a robust and generalized association on athletes’ well-being. This result aligns with the literature that describes the coach–athlete relationship as inherently purposeful [5, 19] and highlights the overall quality of the relationship as fundamental for well-being [20]. Complementarity, as a dimension linked to fluid and effective collaboration, is potentially associated with the satisfaction of an athlete’s psychological need for competence [16]. When a dyad works in harmony to achieve goals, athletes may perceive personal progress and efficacy, which is positively associated with their well-being.

As seen previously, McGee and DeFreese [36] reported an ambivalent association of complementarity, relating it with both engagement and dedication (positive) and emotional and physical exhaustion (negative). The authors speculated that the effort to maintain a highly cooperative relationship could be exhausting. The absence of this negative association in our study may be attributed to contextual differences. Whereas the sample from McGee and DeFreese [36] consisted of college athletes subject to multiple competitive and academic pressures, our sample consisted of adolescents. For this younger group, whose sources of stress are generally of a lower magnitude, the benefits of a cooperative relationship may be more salient than the “price” of the effort to maintain it, which provides a rationale for why complementarity showed a consistently positive association with well-being.

Furthermore, the absence of a significant association between complementarity and life skills is a key empirical finding that confronts initial logical assumptions. While one might expect that the behavioral coordination and fluid interaction inherent in complementarity would naturally foster skills such as teamwork, communication, or problem-solving, our results suggest that this interaction alone does not drive skill acquisition [7, 35]. While complementarity is consistently associated with well-being in the present study, its lack of association between complementarity and life skills indicates that adolescent athletes may position relationships as a functional and task-specific requirement for performance rather than a vehicle for broader personal growth [8, 35].

In contrast, the significant role of commitment in our model suggests that psychological attachment and the intention to maintain the relationship are more critical for the internalization of life skills than immediate behavioral cooperation [10, 37]. These results indicate that the development of life skills depends more on the enduring bond represented by commitment and specific pedagogical actions that transcend the general relational climate [35]. This suggests that the “intention to stay” (commitment) provides the stable cognitive framework necessary for an athlete to translate sporting experiences into transferable life competencies—a process that is not inherently ensured by dyadic synergy and/or sporting proficiency alone.

### Direct association of commitment with life skills

The results demonstrated that commitment in the coach–athlete relationship showed an indirect association with greater well-being through the perceived acquisition of several life skills: goal setting, social skills, leadership, and interpersonal communication. Conversely, an unexpected associative path was observed, where commitment, when analyzed through the mediation of emotional skills, was associated with lower well-being. The absence of studies investigating this complete statistical mediation chain makes direct comparison challenging. However, the literature offers a strong theoretical foundation for understanding the observed connections.

The literature suggests that an athlete’s commitment does not operate in a vacuum but actively shapes the coach’s behavior. As highlighted by Jowett and Shanmugam [19], when athletes demonstrate high commitment and build a quality relationship, this is often associated with coaches focusing more on skill development and learning and exhibiting more positive coaching behaviors. The central theoretical explanation for this dynamic appears to be reciprocity: an athlete who shows commitment inspires in the coach the duty or motivation to reciprocate with high-quality attention, feedback, and instruction [19]. We discuss the association between commitment and each skill sequentially.

### Mediating role of goal setting on well-being

Our results indicate a positive indirect association between commitment and well-being through perceived acquisition of goal setting. This finding is consistent with the literature, which shows that commitment to future goals are associated to life skill transfer [7] and that goal-focused programs can enhance this competence [38]. However, the literature also warns about the sustainability of these achievements, highlighting that these outcomescan be transient [2]. The critical factor for long-term success appears to be the presence of a collaborative and continuous monitoring process, as goals set in isolation are associated with failure and frustration. Therefore, although goal setting is an important mediator, its association with well-being sustainably is potentially linked to a structured support system—that is organized and continuous support from coaches, sports psychologists, and mental performance consultants—that goes beyond the mere formulation of objectives.

### Mediating role of emotional skills on well-being

Our results indicated that commitment is positively associated with emotional skills; however, these life skills are negatively associated with all three well-being domains. The literature on life skills sometimes presents unstable results, and a possible explanation is methodological. A previous study that also reported counterintuitive results was that of Cronin and Allen [13]. Although their findings should be viewed with caution due to the use of analyses based on observed variables (e.g., the PROCESS macro), which do not control for measurement error, it is notable that, even with this limitation, their results already pointed to a negative trend for emotional skills in different models (assessing self-esteem, positive affect, and life satisfaction). In contrast, the present study used SEM with latent variables, which isolates measurement error and allows for a more robust and precise test of the associations, increasing confidence in the findings [29]. By correcting for the attenuation of associations linked to error, SEM increases statistical power and offers more rigorous control of multicollinearity, allowing for a more precise estimation of the unique indirect associations of each skill and providing a more robust test of the proposed model [29].

With the methodological robustness established, the explanation for the negative associations of emotional skills with well-being appears to be theoretical. Indeed, the literature corroborates this finding, showing that negative links between of emotional skills and well-being are found specifically in populations facing adversity [12, 39]. This suggests that emotional self-regulation may be a reactive skill developed out of necessity to withstand difficult contexts [12]. Therefore, high emotional skill in the tested model may not be the cause of low well-being but rather a marker that the athlete has been exposed to a greater number of adverse situations (e.g., injuries, pressure), which are associated with lower well-being and, simultaneously, force the development of an emotional regulation repertoire to cope with them [39].

This phenomenon is described by Ault et al. [12] as “development through adverse experiences,” in which the athlete is forced into a “learning by necessity” to overcome challenges, often focusing more on “surviving” than on “thriving.” The experience of an athlete reported by Pierce et al. [39] illustrates this process: “I started being injured every soccer season. I couldn’t stay healthy. [.]. So, I started to learn how to just be a good teammate [.] but to come to every practice and every game still [.]. Even though that’s not where I wanted to be, it was the only way I could help out the team” [39]. This vignette illustrates how the adversity of the injury is linked to the need to utilize emotional skills while the athlete operates in a psychologically costly motivational state, explaining the coexistence of high skill scores and reduced well-being.

### Mediating role of social skills on emotional well-being

Commitment in the coach–athlete relationship showed a positive indirect association with emotional well-being through the perceived acquisition of the athlete’s social skills. A committed relationship is characterized by an environment of reciprocal investment, in which coaches show a higher likelihood of adopting transformational leadership behaviors and effective communication [6, 40]. As a model of effective communication, a safe space may be identified that is linked to athletes reporting more frequent interactions [5]. In turn, the levels of these skills are associated with the athlete’s perceived participation in the coconstruction of a psychologically safe environment, where open and constructive communication minimizes interpersonal anxiety and improves emotional well-being [6, 37].

### Mediating role of leadership on social well-being

A noteworthy result was the indirect association between coach–athlete commitment and social well-being through leadership. This finding suggests that the quality of the dyad is linked to the athlete’s propensity to positively influence their team. Supported by transformational leadership theory, this association is theoretically identified when coaches in high-quality relationships show mentoring behaviors, such as individualized support and intellectual stimulation [6]. Within this relational context, the athlete is not identified as a passive recipient of instructions. In contrast, they are encouraged to think critically, assume responsibilities, and engage with the team’s values. This process goes beyond mere observation, as a committed coach actively creates opportunities for the athlete to apply and test their skills in practical situations. Therefore, the sport experience can be characterized as a leadership “internship”, in which the athlete learns to set standards and motivate others through observation and experimentation.

By applying their leadership to promote open communication and supporting a climate where “everyone’s voice is heard,” athlete leaders may actively contribute to a climate of psychological safety on the team [37]. This safe environment can strengthen cohesion, reduce conflict, and make members feel more valued [6]. Through these interpersonal dynamics,, athletes may improve the group’s dynamics and solidify their own social position, potentially generating respect and strengthening friendships. This dynamic can be seen as a virtuous cycle: the athlete, empowered by the committed relationship with the coach, applies their new leadership to the team; with improves the group’s dynamics, which, in turn, reinforces the athlete’s own sense of integration and social satisfaction.

### Mediating role of interpersonal communication on psychological well-being

Finally, commitment to the coach–athlete relationship showed a positive indirect association with greater psychological well-being through strengthening the athlete’s interpersonal communication. A committed relationship is linked to the perception ofthe necessary security for the athlete to communicate openly and constructively, within an environment where the interaction is perceived as supported [5, 40]. As highlighted by Jones and Lavallee [41], both athletes and coaches perceive communication as essential for progress in all areas of life, from friendships to career opportunities. In turn, mastery of communication could allow athletes to articulate their needs and collaborate effectively, satisfying their needs for competence and autonomy, which are the essence of psychological well-being [6]. For example, by speaking clearly and paying attention to what the coach is saying and to their body language, the athlete can participate more constructively, knowing that the interaction will be supported [40]. In practice, effective communication is the behavior that allows the athlete to extract the “received support” from the relationship, completing the cycle that promotes their psychological well-being [40].

It is interesting to note, however, a nuance in our findings compared with Jowett’s theoretical proposition. The author, a creator of the 3Cs model, suggested that communication is a tool to improve closeness and complementarity [19]. In this view, communication is the means to achieve better relational quality [19]. However, in the present study, commitment functioned as the antecedent to the development of interpersonal communication (and other skills). This finding does not necessarily contradict Jowett’s model but suggests a more complex dynamic. It is plausible that a baseline level of commitment is necessary for adolescent athletes to feel willing to communicate openly and effectively. Once this communication is established, it may then reinforce other dimensions of the relationship, such as closeness and complementarity, in a virtuous cycle.

The main practical implication is the recommendation to empower coaches with both relational and teaching competencies to support high-quality relationships and the positive development of athletes [5]. Since the results highlight commitment as the key relational dimension for the perceived acquisition of life skills, strategies should focus on strengthening this relational bond. Following Jowett and Shanmugam’s [19] findings, coaches can develop individual plans that project a future for each athlete. They should also structure goal-based programs that involve athletes in decision-making and grant them autonomy, while using continuous communication to ensure that each individual feels valued [2, 19].

For skill development to be effective, coaches must be intentional in their approach, moving beyond implicit learning. This involves planning explicit activities, leveraging “teachable moments” [3], and using pragmatic tools such as logbooks or goal worksheets [38]. These practices are important for transforming relational quality into concrete developmental outcomes. Lastly, coaches should approach the development of emotional skills with particular care, using additional tools to gain a complete understanding of the athlete’s needs and well-being state.

### Suggested teaching priorities for life skills

Other practical implications of this study can inform life skills teaching strategies by offering an empirical guide for prioritizing which competencies to emphasize. Existing pedagogical models, such as that of Ciampolini et al. [3], provide “how” to teach, but the decision of “what” to teach first is often subjective. Our results seem to fill this gap, suggesting that the construction of the relationship itself acts as a structural framework for this process, where closeness precedes commitment, and both serve as a foundation for effective complementarity, supported by models like the Pyramid of Teaching Success in Sport [21].

Based on the magnitude of the associations observed, we propose a tentative pedagogical prioritization. Goal setting could be introduced early, given its robust and positive link to well-being. Next, despite their complex association, emotional skills emerge as a priority for managing the demands of commitment and addressing the potential risks of negative environments. The subsequent focus on social skills, Interpersonal communication, and leadership would align with their respective associations with well-being domains. Other life skills, such as teamwork, problem-solving and decision-making, and time management, could then be integrated. It is essential to emphasize that this order be seen as a flexible guideline, adapted to each context [3, 7]. By proposing an evidence-based framework, this study provides a starting point for future longitudinal investigations into developmental trajectories in sports.

### Strengths and limitations

Among the strengths of this study are its methodological rigor, its use of Structural Equation Modeling, which controls for measurement error, and its theoretical contribution in testing the indirect associations of life skills. However, a limitation involves the global fit indices of the final structural model, which remained borderline. These values suggest that the final model represents a reasonable rather than a perfect approximation of the data. Therefore, while the individual structural paths demonstrated stability and consistency, the results should be interpreted with caution regarding the model’s global fit.

Additionally, the study offers practical implications by proposing a teaching sequence for skills on the basis of the significance and magnitude of the associations identified. However, the study has limitations, such as its cross-sectional design, which precludes definitive causal inferences, and its exclusive focus on the athlete’s perspective, disregarding the views of other agents (e.g., coaches, family, psychologists). Additionally, we did not evaluate the intentionality of teaching life skills by the coaches of the athletes in the sample. We also acknowledge conceptual limitations, such as the recent critiques of the life skills framework (e.g., the absence of a social justice perspective) and the failure to consider the athletes’ own preferences on which skills to prioritize, a factor that could influence their motivation to learn [8, 9, 38].

## Conclusion

The findings of this study demonstrate that life skills represent a significant mechanism in the association between the coach–athlete relationship and athlete well-being. Our results identify a specialized relational dynamic where commitment emerges as the primary correlate for life skill acquisition. Closeness showed a direct association with psychological well-being, while complementarity is directly associated with all domains of well-being. Notably, while goal setting stands out as a robust positive correlate of well-being, the negative association found for emotional skills suggests that their development may serve as a psychological marker of exposure to competitive adversity.

The contribution of this study is twofold. Theoretically, it reveals that the dimensions of the coach–athlete relationship possess functional specificity meaning they relate to developmental outcomes in distinct ways rather than as a unitary construct. Practically, our results suggest that sport-based interventions should prioritize the development of commitment as a foundational prerequisite for effectively teaching life skills. Furthermore, fostering youth’s well-being appears to transcend simply teaching of skills contributing to the stability and quality of the relational bond.Future research could include the coach’s perspective to obtain a more comprehensive view of the relationship and utilize longitudinal designs to observe the transition from implicit to explicit life skills teaching. Other agents could be investigated, such as parents, sport psychologists, and social workers, for a broader view of the influences on athletes. Furthermore, investigating other potential statistical mediators, such as mental performance skills (e.g., motivation, resilience) and the motivational climate, would provide a more holistic understanding of the pathways to PYD in youth sport.

## Supplementary Information

Below is the link to the electronic supplementary material.

Supplementary Material 1

Supplementary Material 2

## Data Availability

The data that support the findings of this study are available upon request from the corresponding author, W.R.T. The data are not publicly available owing to ethical restrictions, as they contain sensitive information from adolescent participants.

## Declarations

### Declarations of generative AI and AI-assisted technologies

During the preparation of this work, the authors used Gemini (Google) and Curie (Springer Nature) to improve language clarity and readability. After using these tools/services, the authors reviewed and edited the content as needed and take full responsibility for the content of the published article.
