# Supplementary material for: The mediating role of life skills in the association of the coach–athlete relationship with youth athletes’ well-being
Source: Discov Ment Health. 2026 May 3;6(1):116. doi: 10.1007/s44192-026-00468-7 (PMC13280274; doi:10.1007/s44192-026-00468-7)
Supplement: Supplementary file 1 — Supplementary Material 1 [file 44192_2026_468_MOESM1_ESM.docx]

**# Model 1 - Basic (General Factors)**

Model1 <- '

# Structural Model (Regressions)

# Predicting General Well-being

Well_Being_General ~ b1*Life_Skills_General + c1*CART_General + Region + Sport_Type + Sex + Age + Time_Practicing

# Predicting Life Skills

Life_Skills_General ~ a1*CART_General + Region + Sport_Type + Sex + Age + Time_Practicing

# Predicting Coach-Athlete Relationship (Control)

CART_General ~ Region + Sport_Type + Sex + Age + Time_Practicing

# Measurement Model (Second-Order Factors)

# Life Skills (8 dimensions loading onto General Factor)

Life_Skills_General =~ Teamwork + Goal_Setting + Social_Skills + Problem_Solving + Emotional_Skills + Leadership + Time_Management + Interpersonal_Comm

# Well-being (3 dimensions loading onto General Factor)

Well_Being_General =~ Psychological_WB + Social_WB + Emotional_WB

# Coach-Athlete Relationship (3 dimensions loading onto General Factor)

CART_General =~ Closeness + Commitment + Complementarity

# Measurement Model (First-Order Factors - Indicators)

# Life Skills

Teamwork =~ HV_1 + HV_2 + HV_3 + HV_4 + HV_5 + HV_6 + HV_7

Goal_Setting =~ HV_8 + HV_9 + HV_10 + HV_11 + HV_12 + HV_13 + HV_14

Social_Skills =~ HV_15 + HV_16 + HV_17 + HV_18 + HV_19

Problem_Solving =~ HV_20 + HV_21 + HV_22 + HV_23

Emotional_Skills =~ HV_24 + HV_25 + HV_26 + HV_27

Leadership =~ HV_28 + HV_29 + HV_30 + HV_31 + HV_32 + HV_33 + HV_34 + HV_35

Time_Management =~ HV_36 + HV_37 + HV_38 + HV_39

Interpersonal_Comm =~ HV_40 + HV_41 + HV_42 + HV_43

# Well-being

Psychological_WB =~ csme_9 + csme_10 + csme_11 + csme_12 + csme_13 + csme_14

Social_WB =~ csme_4 + csme_5 + csme_6 + csme_7 + csme_8

Emotional_WB =~ csme_1 + csme_2 + csme_3

# Coach-Athlete Relationship

Closeness =~ cartq_atleta_3 + cartq_atleta_5 + cartq_atleta_8 + cartq_atleta_9

Commitment =~ cartq_atleta_1 + cartq_atleta_2 + cartq_atleta_6

Complementarity =~ cartq_atleta_4 + cartq_atleta_7 + cartq_atleta_10 + cartq_atleta_11

'

**# Model 2 - Initial (Specific Dimensions)**

Model2 <- '

# Regressions: Predicting Well-Being Dimensions

# Emotional Well-being

Emotional_WB ~ Closeness + Commitment + Complementarity +

Teamwork + Goal_Setting + Social_Skills +

Problem_Solving + Emotional_Skills + Leadership +

Time_Management + Interpersonal_Comm +

Region + Sport_Type + Sex + Age + Time_Practicing

# Social Well-being

Social_WB ~ Closeness + Commitment + Complementarity +

Teamwork + Goal_Setting + Social_Skills +

Problem_Solving + Emotional_Skills + Leadership +

Time_Management + Interpersonal_Comm +

Region + Sport_Type + Sex + Age + Time_Practicing

# Psychological Well-being

Psychological_WB ~ Closeness + Commitment + Complementarity +

Teamwork + Goal_Setting + Social_Skills +

Problem_Solving + Emotional_Skills + Leadership +

Time_Management + Interpersonal_Comm +

Region + Sport_Type + Sex + Age + Time_Practicing

# Regressions: Predicting Life Skills (Mediators)

Teamwork ~ Closeness + Commitment + Complementarity + Region + Sport_Type + Sex + Age + Time_Practicing

Goal_Setting ~ Closeness + Commitment + Complementarity + Region + Sport_Type + Sex + Age + Time_Practicing

Social_Skills ~ Closeness + Commitment + Complementarity + Region + Sport_Type + Sex + Age + Time_Practicing

Problem_Solving ~ Closeness + Commitment + Complementarity + Region + Sport_Type + Sex + Age + Time_Practicing

Emotional_Skills ~ Closeness + Commitment + Complementarity + Region + Sport_Type + Sex + Age + Time_Practicing

Leadership ~ Closeness + Commitment + Complementarity + Region + Sport_Type + Sex + Age + Time_Practicing

Time_Management ~ Closeness + Commitment + Complementarity + Region + Sport_Type + Sex + Age + Time_Practicing

Interpersonal_Comm ~ Closeness + Commitment + Complementarity + Region + Sport_Type + Sex + Age + Time_Practicing

# Regressions: Control variables on Coach-Athlete Relationship

Closeness ~ Region + Sport_Type + Sex + Age + Time_Practicing

Commitment ~ Region + Sport_Type + Sex + Age + Time_Practicing

Complementarity ~ Region + Sport_Type + Sex + Age + Time_Practicing

# Measurement Model (CFA definitions)

# Life Skills

Teamwork =~ HV_1 + HV_2 + HV_3 + HV_4 + HV_5 + HV_6 + HV_7

Goal_Setting =~ HV_8 + HV_9 + HV_10 + HV_11 + HV_12 + HV_13 + HV_14

Social_Skills =~ HV_15 + HV_16 + HV_17 + HV_18 + HV_19

Problem_Solving =~ HV_20 + HV_21 + HV_22 + HV_23

Emotional_Skills =~ HV_24 + HV_25 + HV_26 + HV_27

Leadership =~ HV_28 + HV_29 + HV_30 + HV_31 + HV_32 + HV_33 + HV_34 + HV_35

Time_Management =~ HV_36 + HV_37 + HV_38 + HV_39

Interpersonal_Comm =~ HV_40 + HV_41 + HV_42 + HV_43

# Well-Being

Psychological_WB =~ csme_9 + csme_10 + csme_11 + csme_12 + csme_13 + csme_14

Social_WB =~ csme_4 + csme_5 + csme_6 + csme_7 + csme_8

Emotional_WB =~ csme_1 + csme_2 + csme_3

# Coach-Athlete Relationship

Closeness =~ cartq_atleta_3 + cartq_atleta_5 + cartq_atleta_8 + cartq_atleta_9

Commitment =~ cartq_atleta_1 + cartq_atleta_2 + cartq_atleta_6

Complementarity =~ cartq_atleta_4 + cartq_atleta_7 + cartq_atleta_10 + cartq_atleta_11

'
