# Supplementary material for: The mediating role of life skills in the association of the coach–athlete relationship with youth athletes’ well-being
Source: Discov Ment Health. 2026 May 3;6(1):116. doi: 10.1007/s44192-026-00468-7 (PMC13280274; doi:10.1007/s44192-026-00468-7)
Supplement: Supplementary file 2 — Supplementary Material 2 [file 44192_2026_468_MOESM2_ESM.docx]

| **Supplementary table 1**. Direct paths adjusted through the structural equation model between the analyzed variables at model 2. | | | | | |  |
| --- | --- | --- | --- | --- | --- | --- |
| **Direct paths** | **β** | **95% CI** | **SE** | **β stan.** | **P-value** | |
| **Emotional Well-Being** |  |  |  |  |  | |
| Goal Setting | 0.424 | (0.254/0.595) | 0.087 | 0.831 | <0.001 | |
| Problem Solving | -0.094 | (-0.33/0.142) | 0.12 | -0.195 | 0.433 | |
| Time Management | -0.051 | (-0.199/0.096) | 0.075 | -0.111 | 0.494 | |
| Emotional Skills | -0.341 | (-0.535/-0.147) | 0.099 | -0.632 | 0.001 | |
| Teamwork | -0.049 | (-0.221/0.122) | 0.088 | -0.078 | 0.574 | |
| Leadership | -0.256 | (-0.74/0.228) | 0.247 | -0.518 | 0.300 | |
| Social Skills | 0.313 | (0.13/0.496) | 0.093 | 0.496 | 0.001 | |
| Inter. Communication | -0.139 | (-0.454/0.176) | 0.161 | -0.342 | 0.388 | |
| Closeness | -0.081 | (-0.335/0.173) | 0.13 | -0.15 | 0.531 | |
| Commitment | 0.137 | (-0.053/0.327) | 0.097 | 0.191 | 0.157 | |
| Complementarity | 0.479 | (0.29/0.664) | 0.096 | 0.624 | <0.001 | |
| **Social Well-Being** |  |  |  |  |  | |
| Goal Setting | 0.277 | (0.141/0.442) | 0.077 | 0.367 | <0.001 | |
| Problem Solving | -0.131 | (-0.411/0.149) | 0.143 | -0.174 | 0.358 | |
| Time Management | -0.092 | (-0.261/0.077) | 0.086 | -0.125 | 0.286 | |
| Emotional Skills | -0.434 | (-0.689/-0.26) | 0.077 | -0.546 | <0.001 | |
| Teamwork | -0.139 | (-0.361/0.082) | 0.113 | -0.14 | 0.219 | |
| Leadership | 0.289 | (0.157/0.694) | 0.136 | 0.356 | 0.021 | |
| Social Skills | -0.095 | (-0.394/0.204) | 0.152 | -0.133 | 0.533 | |
| Inter. Communication | -0.049 | (-0.347/0.249) | 0.152 | -0.077 | 0.746 | |
| Closeness | -0.371 | (-1.466/0.724) | 0.559 | -0.441 | 0.507 | |
| Commitment | 0.925 | (0.248 / 1.601) | 0.345 | 0.491 | 0.007 | |
| Complementarity | 0.627 | (0.423 / 0.832) | 0.104 | 0.553 | <0.001 | |
| **Psychological Well-Being** |  |  |  |  |  | |
| Goal Setting | 0.353 | (0.247/0.457) | 0.053 | 0.563 | <0.001 | |
| Problem Solving | -0.249 | (-0.663/0.165) | 0.211 | -0.319 | 0.239 | |
| Time Management | 0.044 | (-0.042/0.131) | 0.044 | 0.06 | 0.316 | |
| Emotional Skills | -0.242 | (-0.361/-0.113) | 0.063 | -0.366 | <0.001 | |
| Teamwork | -0.003 | (-0.159/0.153) | 0.08 | -0.003 | 0.967 | |
| Leadership | -2.147 | (-5.476/1.182) | 1.699 | -2.659 | 0.206 | |
| Social Skills | 0.11 | (-0.274/0.494) | 0.196 | 0.154 | 0.573 | |
| Inter. Communication | 0.359 | (0.25/0.462) | 0.055 | 0.553 | <0.001 | |
| Closeness | 0.253 | (0.174/0.344) | 0.042 | 0.402 | <0.001 | |
| Commitment | -0.018 | (-0.697/0.661) | 0.346 | 4.823 | 0.959 | |
| Complementarity | 0.516 | (0.341/0.691) | 0.089 | 0.549 | <0.001 | |
| **Life Skills** |  |  |  |  |  | |
| **Goal Setting** |  |  |  |  |  | |
| Closeness | -0.044 | (-0.186/0.097) | 0.072 | -0.047 | 0.541 | |
| Commitment | 2.014 | (1.259/2.768) | 0.385 | 0.807 | <0.001 | |
| Complementarity | 0.278 | (-0.206/0.762) | 0.247 | 0.242 | 0.261 | |
| **Problem Solving** |  |  |  |  |  | |
| Closeness | -0.082 | (-0.201/0.037) | 0.061 | -0.073 | 0.175 | |
| Commitment | 1.887 | (1.119/2.656) | 0.392 | 0.893 | <0.001 | |
| Complementarity | 0.108 | (-0.109/0.326) | 0.111 | 0.08 | 0.329 | |
| **Time Management** |  |  |  |  |  | |
| Closeness | -0.127 | (-0.545/0.291) | 0.213 | -0.11 | 0.551 | |
| Commitment | 1.673 | (0.972/2.373) | 0.357 | 0.828 | <0.001 | |
| Complementarity | 0.232 | (-0.174/0.638) | 0.207 | 0.167 | 0.263 | |
| **Emotional Skills** |  |  |  |  |  | |
| Closeness | -0.081 | (-0.23/0.068) | 0.076 | -0.07 | 0.289 | |
| Commitment | 1.92 | (1.216/2.623) | 0.359 | 0.813 | <0.001 | |
| Complementarity | 0.19 | (-0.121/0.5) | 0.158 | 0.139 | 0.231 | |
| **Teamwork** |  |  |  |  |  | |
| Closeness | 0.015 | (-0.205/0.234) | 0.112 | 0.018 | 0.897 | |
| Commitment | 1.174 | (0.705/1.643) | 0.239 | 0.901 | <0.001 | |
| Complementarity | 0.07 | (-0.11/0.251) | 0.092 | 0.069 | 0.446 | |
| **Leadership** |  |  |  |  |  | |
| Closeness | -0.029 | (-0.115/0.057) | 0.044 | -0.027 | 0.512 | |
| Commitment | 2.221 | (1.28/2.761) | 0.379 | 0.849 | <0.001 | |
| Complementarity | 0.067 | (-0.096/0.23) | 0.083 | 0.053 | 0.423 | |
| **Social Skills** |  |  |  |  |  | |
| Closeness | -0.206 | (-0.649/0.236) | 0.226 | -0.174 | 0.361 | |
| Commitment | 2.067 | (1.363/2.848) | 0.375 | 0.942 | <0.001 | |
| Complementarity | 0.107 | (-0.206/0.419) | 0.159 | 0.075 | 0.504 | |
| **Interpersonal Communication** |  |  |  |  |  | |
| Closeness | -0.095 | (-0.233/0.042) | 0.07 | -0.072 | 0.173 | |
| Commitment | 2.596 | (1.672/3.520) | 0.471 | 0.94 | <0.001 | |
| Complementarity | 0.196 | (-0.204/0.596) | 0.204 | 0.122 | 0.337 | |
| Notes: β = Non-standardized coefficient. β Stand= Standardized coefficient. The analysis was adjusted for sex, age, region, type of sport and time practicing the sport. | | | | | | |
